# Case Report: EPD-assisted “Open Umbrella” retrieval of a migrated traumatic intravascular foreign body

**DOI:** 10.3389/fmed.2026.1802337

**Published:** 2026-04-28

**Authors:** Wei-Han Luo, Ting Zhu, Teng-Chao Zhou, Jie Tan, Wen-Bo Liu

**Affiliations:** 1 Department of Interventional Vascular Surgery, The Central Hospital of Shaoyang, Shaoyang, China; 2 School of Nursing, Shaoyang University, Shaoyang, China; 3 Department of Interventional Vascular Surgery, Hunan Provincial People's Hospital (The First Affiliated Hospital of Hunan Normal University), Changsha, China

**Keywords:** “Open Umbrella” technique, damage control interventional radiology, embolic protection device, femoral artery, intravascular foreign body

## Abstract

Intravascular foreign bodies (IFBs) resulting from penetrating trauma pose significant clinical challenges due to their irregular geometry, thrombogenicity, and propensity for distal migration. While endovascular snare retrieval is the established standard for smooth, tubular iatrogenic objects, it often fails when fragments lie flush against the vessel wall. We report the case of a 71-year-old male who sustained a penetrating injury to the left femoral artery by a metallic fragment. During initial surgical repair at a local hospital, the fragment migrated distally. Computed tomography angiography localized the triangular foreign body within the superficial femoral artery. We utilized an “Open Umbrella” technique, repurposing a distal embolic protection device (EPD) as an active capture tool. This methodology facilitated successful retrieval while providing continuous distal perfusion and obligate protection against secondary embolization (“trash foot” phenomenon). The procedure adhered to the principles of Damage Control Interventional Radiology (DCIR), avoiding complex surgical re-exploration. Our experience highlights that EPD-assisted retrieval offers a bimodal advantage of high-probability capture and embolic prophylaxis. For successful extraction, we recommend the “2-Fr rule”—utilizing a retrieval sheath at least 2-Fr larger than the object's diameter. EPD-assisted retrieval should be considered a primary salvage or even a first-line strategy for complex traumatic IFBs.

## Introduction

Intravascular foreign bodies (IFBs) constitute an uncommon but potentially fatal complication arising from both iatrogenic medical procedures and penetrating trauma (1). Iatrogenic sources, such as fractured guidewires and catheters, constitute the majority of cases (2). However, traumatic IFBs, such as bullets or industrial metallic shards, present distinct challenges due to their irregular geometry, thrombogenic potential, and lack of standardized retrieval protocols (3). Since the initial description of percutaneous retrieval in 1964, the management paradigm for IFBs has shifted markedly from open surgical extraction toward minimally invasive endovascular retrieval techniques, which are now widely regarded as the standard of care (4, 5).

Unretrieved IFBs are associated with high complication rate, including sepsis, perforation, and distal ischemia. While the success rate of percutaneous retrieval exceeds 90%, traditional devices like snares require a “free end” to grasp, which is often absent in traumatic fragments (6, 7). This case reports the novel application of distal embolic protection devices (EPDs), typically reserved for carotid stenting or TAVI, as active retrieval mechanisms for complex traumatic debris.

## Case presentation

A 71-year-old Asian male, with no significant past medical or surgical history, presented with an acute penetrating injury to the left medial thigh caused by a metal fragment while chopping firewood. Initial radiography at a local hospital identified a punctate high-density foreign body (FB) in the soft tissue, with no associated fracture (Day 0). Emergency exploration revealed a left femoral artery rupture; vascular anastomosis was performed. Post-anastomosis, the left lower limb remained hemodynamically stable with palpable distal pulses, and no immediate signs of acute limb ischemia (such as pallor or paresthesia) were noted. However, during the repair, a metallic FB was observed to have migrated distally within the arterial lumen during intraoperative digital radiography (DR). Recognizing the imminent risk of secondary distal embolization, vascular occlusion, or endothelial trauma posed by the sharp fragment, the patient was urgently transferred to our tertiary institution for specialized endovascular management following initial stabilization.

On admission, physical examination revealed limited movement of the left lower limb. The left thigh wound was covered with a sterile dressing. No edema was observed in either lower extremity. Bilateral femoral and dorsalis pedis pulses were palpable and symmetrical. Neurological examination showed that physiological reflexes were present, and no pathological reflexes were elicited. Computed tomography angiography (CTA) of the lower extremity arteries demonstrated a retained intravascular foreign body within the left superficial femoral artery, with preserved distal arterial flow (Day 1). Based on these findings, a diagnosis of left femoral artery injury complicated by a retained intravascular foreign body was established.

Endovascular retrieval of the foreign body was planned to prevent distal embolization and vascular occlusion. The procedure was performed in a digital subtraction angiography suite with the patient in the supine position under local anesthesia. Vascular access was obtained via the right common femoral artery, and a 6F sheath was inserted. A contralateral crossover approach was intentionally selected over an ipsilateral antegrade approach to avoid instrumentation and manual compression near the fresh surgical anastomosis on the left femoral artery, thereby minimizing the risk of mechanical disruption or site-specific infection. Angiography revealed a patent left superficial femoral artery with a triangular-shaped foreign body located in the mid-to-distal segment, while distal arteries remained well opacified.

The fragment was cautiously crossed with a 0.014-inch guidewire (Command; Abbott Vascular, Santa Clara, CA). Subsequently, a SpiderFX EPDs (Medtronic, Minneapolis, MN) was deployed distal to the IFB to act as an active capture tool. Initial attempts to retrieve the fragment through the 6F sheath were unsuccessful due to the combined bulk of the EPD and the entrapped metal fragment. The 6F sheath was subsequently exchanged for an 8F retrieval sheath (Cook Medical, Bloomington, IN), allowing secure fixation and complete removal of the foreign body. Repeat angiography confirmed good arterial patency, smooth blood flow, and absence of contrast extravasation or residual stenosis. A total of 120 mL of contrast agent was used. The arterial access site was closed using a vascular closure device.

**Figure 1:**
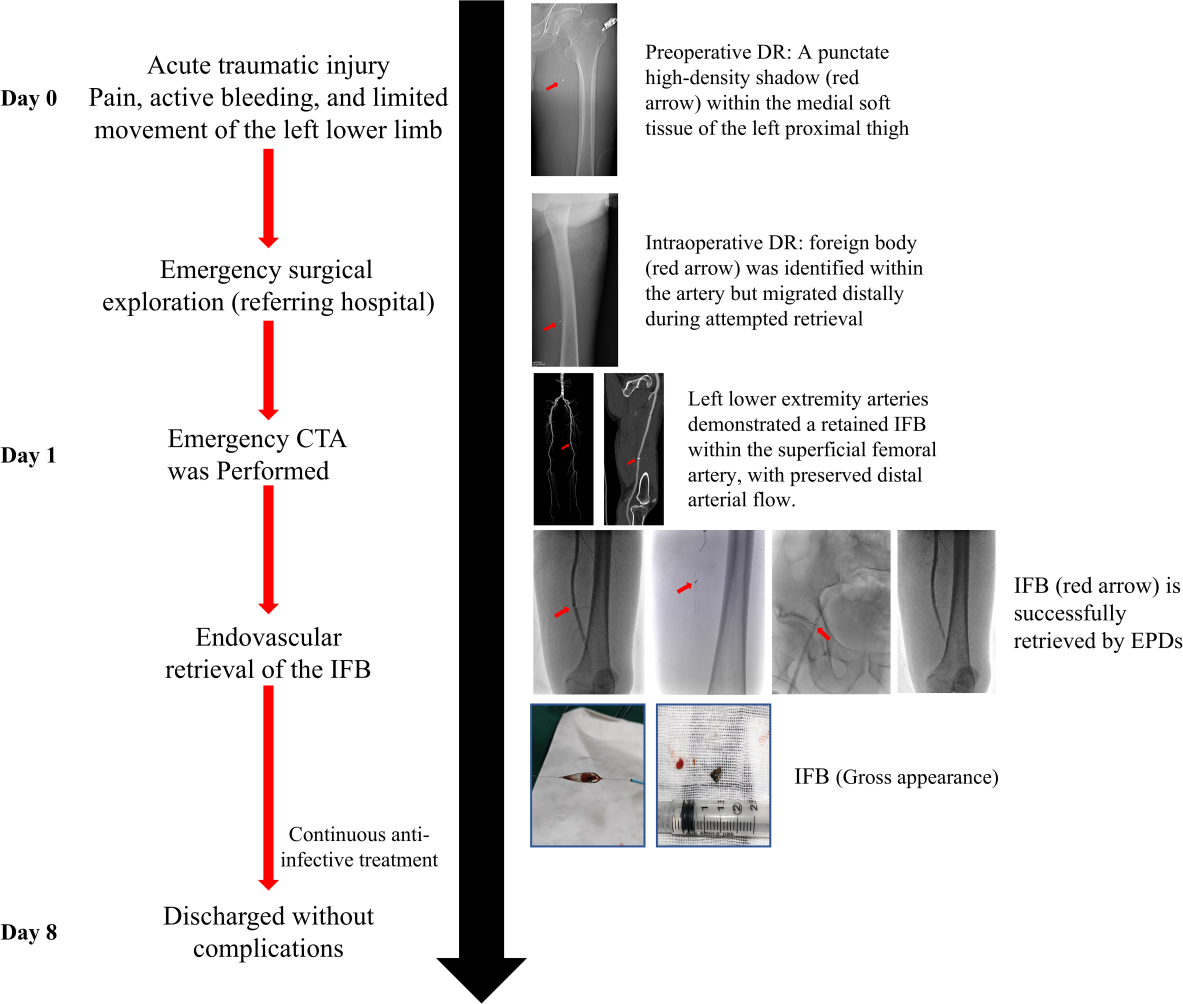
Clinical timeline illustrating the patient's diagnostic journey, therapeutic interventions, and outcomes, in accordance with the CARE guidelines.

The procedure was completed without complications. Postoperatively, the patient was instructed to maintain immobilization of the right lower limb for 12 h, and close monitoring of limb perfusion and puncture-site bleeding was conducted. The patient recovered uneventfully, with no evidence of distal ischemia or recurrent vascular injury during the immediate postoperative period. Written informed consent was obtained for the procedure and for publication of this case report. Figure 1 illustrates the chronological timeline of the patient's diagnosis, treatments, and clinical outcomes in accordance with the CARE guidelines.

## Discussion

The migration of traumatic metallic fragments within the arterial system poses a significant clinical challenge (1, 3). This migration in our case likely resulted from mechanical manipulation during the vascular reconstruction and the subsequent restoration of physiological blood flow following the removal of vascular clamps. Unlike iatrogenic IFBs (e.g., fractured catheters), which are typically smooth and tubular, traumatic IFBs are often irregular, sharp, and highly thrombogenic (8). As demonstrated in this case, the shift from soft tissue to the endovascular lumen complicates retrieval due to the risk of further distal migration and arterial wall injury (9, 10).

While the gooseneck snare remains the established paradigm for endovascular retrieval, its efficacy is inherently limited by the spatial orientation and morphology of the target object (6, 11). Effective snare engagement relies on the presence of a “free-floating” edge to facilitate capture (12). In this case, the triangular, low-profile nature of the metallic fragment made it prone to lying flush against the vessel wall, rendering snare-based “proximal grab” or “lateral grasp” techniques technically difficult and potentially traumatic to the endothelium. Furthermore, aggressive snare manipulation carries a high risk of fragmenting the object or dislodging associated thrombi (13).

Transitioning from its conventional role as a passive embolic filter in procedures such as CAS or TAVI, the EPDs was utilized here in an “Open Umbrella” capacity, effectively repurposing it into an active retrieval tool for complex traumatic IFBs (14, 15). This methodology provides a synergistic advantage of mechanical efficacy and procedural safety. Firstly, the porous mesh maintains continuous distal perfusion while “sieving” the fragment from the bloodstream, thereby neutralizing the hemodynamic drag that frequently precipitates slippage during traditional snare-based maneuvers (14). Furthermore, the device's 360-degree circumferential apposition overcomes the geometric constraints of wall-hugging or irregular fragments, ensuring secure encapsulation regardless of their spatial orientation (16). Crucially, given that traumatic fragments are inherently thrombogenic, the EPDs serves as an intrinsic “safety net” that captures secondary thrombi or “surgical trash” liberated during manipulation. This mitigates the risk of distal small-vessel occlusion, the so-called “trash foot” phenomenon, and significantly elevates the safety profile of the intervention (17).

This case underscores the clinical utility of damage control interventional radiology (DCIR) (18). By prioritizing a minimally invasive percutaneous approach over complex surgical re-exploration, we circumvented the substantial morbidity and infection risks associated with operating in a contaminated traumatic field. The EPD-assisted strategy facilitated “no-touch” isolation and atraumatic retrieval of the IFBs, effectively minimizing further mechanical insult to the already compromised arterial segment (14). Nevertheless, technical success is strictly contingent upon meticulous procedural planning, particularly regarding sheath caliber. Our experience highlights that an initial 6F sheath was insufficient for the collective bulk of the EPD and the entrapped fragment. Consequently, we advocate for the literature-supported “2-Fr rule,” which stipulates that the retrieval sheath should be at least 2-Fr larger than the maximum diameter of the foreign body to ensure seamless withdrawal and preclude potentially catastrophic device entrapment (19).

## Conclusion

The management of distally migrated traumatic IFBs necessitates a versatile and adaptive endovascular armamentarium. While conventional snares remain the standard of care, their efficacy is frequently compromised by the irregular morphology and “wall-hugging” nature of traumatic fragments. This case highlights that EPDs can be effectively repurposed as active retrieval instruments via the “Open Umbrella” technique. This methodology offers a distinct bimodal advantage: high-probability entrapment of complex geometries and obligate prophylaxis against distal embolization. Based on this experience, EPD-assisted retrieval may be considered a valuable adjunct or alternative in selected complex cases where traditional snare-based techniques are likely to fail or pose an unacceptable risk of secondary embolization.

## Data Availability

The original contributions presented in the study are included in the article/supplementary material, further inquiries can be directed to the corresponding author.
